# Expression of the plasminogen system in the physiological mouse ovary and in the pathological polycystic ovary syndrome (PCOS) state

**DOI:** 10.1186/s12958-019-0472-0

**Published:** 2019-03-16

**Authors:** Genia F. Burchall, Dodie S. Pouniotis, Helena J. Teede, Sanjeeva Ranasinha, Kirsty A. Walters, Terrence J. Piva

**Affiliations:** 1 School of Health and Biomedical Sciences, RMIT University, Bundoora, Victoria 3083 Australia; 2 School of Public Health and Preventive Medicine, Monash University, Clayton, Victoria 3168 Australia; 3 School of Women’s & Children’s Health, University of New South Wales, Sydney, NSW 2052 Australia

**Keywords:** Polycystic ovary syndrome, PAI-1, Fibrinolytic/proteolytic markers, Ovulation, Folliculogenesis

## Abstract

**Background:**

The fibrinolytic system and its inhibitors play a number of roles, apart from their function in blood haemostasis and thrombosis, namely in ovarian folliculogenesis and in ovulation. Plasminogen is converted to active plasmin at the time of follicular rupture through a decrease in plasminogen activator inhibitor-1 (PAI-1) and an increase in plasminogen activators. Oligo−/anovulation and follicle arrest are key characteristics of PCOS, but studies evaluating fibrinolytic/proteolytic markers within human or animal PCOS ovaries are lacking. We aimed to investigate and compare the expression and distribution of the plasminogen system markers in PCOS and control ovaries.

**Methods:**

A hyperandrogenised PCOS mouse model was used that mimics the ovarian, endocrine and metabolic features of the human condition. Immunohistochemistry and digital image analysis were used to investigate and compare fibrinolytic/proteolytic markers plasminogen, plasminogen/plasmin, tissue plasminogen activator, urokinase plasminogen activator and inhibitor PAI-1 in PCOS and control ovaries. Student’s t-test was used to compare data sets for normally distributed data and Wilcoxon-Mann Whitney test for non-normally distributed data.

**Results:**

We noted differences in the ovarian distribution of PAI-1 that was expressed throughout the PCOS ovary, unlike the peripheral distribution observed in control ovaries. Plasminogen was present in small follicles only in PCOS ovaries but not in small follicles of control ovaries. When we assessed and compared PAI-1 expression within follicles of different developmental stages we also noted significant differences for both the PCOS and control ovaries. While we noted differences in distribution and expression within specific ovarian structures, no differences were noted in the overall ovarian expression of markers assessed between acyclical PCOS mice and control mice at the diestrus stage of the estrous cycle.

**Conclusions:**

Our novel study, that comprehensively assessed the fibrinolytic/proteolytic system in the mouse ovary, showed the expression, differential localisation and a potential role for the plasminogen system in the physiological mouse ovary and in PCOS. Androgens may be involved in regulating expression of the ovarian plasminogen system. Further studies evaluating these markers at different time-points of ovulation may help to further clarify both physiological and potential pathological actions these markers play in ovulatory processes distorted in PCOS.

## Background

Polycystic Ovary Syndrome (PCOS) is a common condition, affecting 12–18% of reproductive aged women and is diagnosed based on ovulatory and menstrual disturbance, hyperandrogenism and polycystic appearance of ovaries on ultrasound [1–7]. Women with PCOS have increased risk factors for cardiovascular disease (CVD) and venous thromboembolism (VTE) likely relating to the aberrant metabolic features and elevated BMI, frequently present in PCOS, but also to the recently identified abnormalities in the haemostatic and fibrinolytic systems [8, 9]. A mild to moderate hypofibrinolytic state is present in women with PCOS, relating to increased plasminogen activator inhibitor 1 (PAI-1) that prevents conversion of the pro-enzyme plasminogen to its active form of plasmin [9]. The fibrinolytic system markers and its inhibitors play a number of roles, apart from their function in blood haemostasis and thrombosis, namely in ovulation and folliculogenesis. In animal models it was shown that follicle rupture is achieved following an increase in plasminogen activator/s (PA) and a decrease in fibrinolytic inhibitor PAI-1 [10]. Plasmin has also been detected in the ovary of porcine models at the time of or just before rupture of the ovarian follicle [10]. This suggests that plasminogen is converted to its functional form of plasmin at the time of follicular rupture. It was also noted that immediately prior to ovulation PAI-1 levels are at their highest in the centre of the ovary, where immature follicles reside, whereas PA tissue plasminogen activator (tPA) is highest at the surface, where preovulatory follicles are localised [11]. A review by Liu et al concluded that the serine protease tPA as well as inhibitor PAI-1 play the most significant role in ovulation processes but also have a function in oocyte maturation [12]. These researchers also noted interspecies differences in expression of different PA as well as a cell-specific and time-dependent response of the ovarian expression of both tPA and PAI-1. To our knowledge, there have been no published studies that have evaluated the fibrinolytic/proteolytic system in PCOS mouse models. However in a study by Devin et al*,* using transgenic female mice that constitutively secrete a stable variant of active human PAI-1, it was observed that these mice contain many large cystic structures within their ovaries and had plasma testosterone levels nearly twice as high as control mice [11]. They concluded that overexpression of PAI-1 promotes the development of polycystic ovarian changes, however they did not evaluate metabolic or fibrinolytic markers in these mice. Ma et al on the other hand showed that mice which lacked PAI-1, unlike their wild type counterparts, did not develop high fat/high carbohydrate diet-induced obesity and insulin resistance, which are key clinical features of PCOS [13].

Physiological studies in humans have also identified the presence of tPA and PAI-1 in human ovaries [14–16] and only few studies have been carried out evaluating fibrinolytic/proteolytic system markers and inhibitors within human PCOS ovaries. Ambekar et al performed proteomic analysis of ovarian follicular fluid from women with PCOS compared to that of healthy aged-matched non-PCOS women. They found that plasminogen is degraded within the follicular fluid of ovaries in women with PCOS compared to non-PCOS women [17]. Quantitation of overexpressing proteins identified the upregulation of SERPINA 1, a plasminogen activator inhibitor in the follicular fluid of ovaries of females with PCOS that can result in reduced plasmin levels. Atiomo et al found that PAI-1 was expressed in both the granulosa and theca cell compartments of PCOS and non-PCOS human ovaries and while there was overall more PAI-1 detected around the follicles of polycystic ovaries this did not reach statistical significance when compared to control ovaries [16]. While these studies presented interesting findings, they both had small sample sizes and in the latter study [16] samples were taken from women at various stages of the menstrual cycle. In contrast, Devin et al found PAI-1 to be prevalent in all five of the human PCOS ovary specimens, localised to the granulosa cells lining cystic structures and to atretic follicles, but none of the non-PCOS ovaries demonstrated significant PAI-1 expression [11].

Oligo−/anovulation and follicle arrest are key characteristics of PCOS, which are associated with infertility in this group of women, with the exact mechanism/s remaining unclear [5–7, 18, 19]. While PAI-1 is believed to play a physiological role within the ovary in preventing ovulation of immature central follicles, persistent elevation of this fibrinolytic/proteolytic inhibitor, as is noted in the plasma of women with PCOS, may potentially contribute to a lack of ovulation. Plasminogen activator inhibitor-1 may prevent follicular wall breakdown of more mature preovulatory follicles and may contribute to the ovarian architecture currently observed in the ovaries of women with PCOS.

In this context, we aimed to investigate and compare the expression and distribution of fibrinolytic/proteolytic markers: plasminogen, plasmin, PA: tPA and urokinase plasminogen activator (uPA) and inhibitor PAI-1 in control and PCOS ovaries. We used a PCOS mouse model treated with dihydrotestosterone (DHT) that display extensive ovarian, endocrine and metabolic features of humans affected by PCOS including oligo- or anovulation, irregular menstrual cycles, polycystic ovaries, obesity and dislipidaemia [20]. We hypothesized that all fibrinolytic/proteolytic markers investigated in this study will be expressed in both the PCOS and control mice ovaries, highlighting their potential role in either physiological or pathological ovulation (and/or other ovarian functions), however they will vary in their intensity and distribution within the tissues. In general, PAI-1 expression will be elevated in the PCOS mouse ovaries compared to that of controls and this marker will be distributed throughout the tissue, while in controls it will be localised mainly centrally. The ovarian expression and distribution of PA may be similar, but differences may be noted for plasmin/plasminogen in PCOS and control mice. We further hypothesized there will be a gradual increase in PAI-1 expression in both control and PCOS ovaries as follicles develop from small through to antral stages, however a decreased expression of this marker will be noted in preovulatory follicles of normal controls, though not so in the same follicles of PCOS ovaries.

## Methods

### Mice

In this study all mice had a wild-type androgen receptor (AR) genotype and were taken from a colony used to generate AR-knockout mice [21, 22]. This colony has been backcrossed onto a C57BL/6J background for at least 10 generations prior to use in experiments. Littermate controls were used. Mice were fed a standard chow diet (2018S Teklad Global 18% Protein Rodent Diet, ENVIGO, USA), and were maintained under standard housing conditions (ad libitum access to food and water in a temperature and humidity controlled, 12-h light cycle environment) at the ANZAC Research Institute [Concord, Australia] [20]. All procedures were performed under ketamine/xylazine anaesthesia. All procedures were approved by the Sydney Local Health District Animal Welfare Committee within NHMRC guidelines for animal experimentation [20].

### Development of PCOS mouse model

PCOS was induced by postnatal androgenization as previously described [20]. Briefly, 21-day old female mice were implanted sc with either a blank (controls) 1 cm SILASTIC brand implant (id, 1.47 mm; od, 1.95 mm, Dow Corning Corp, catalog no. 508–006), or an implant containing ~ 10 mg DHT. Mice were collected after 13 weeks of drug administration (*n* = 8 control mice; *n* = 9 DHT mice) when the mice were 16 weeks of age [20].

### Assessment of estrous cycle

The stage of the estrous cycle was identified in the mice on a daily basis using light microscopy examination of vaginal epithelial cell smears, as previously described [20]. The stage of the estrous cycle was determined based on the presence or absence of leukocytes, cornified epithelial cells, and nucleated epithelial cells. The proestrous stage was characterized by the presence of mostly nucleated and some cornified epithelial cells; at the estrous stage mostly cornified epithelial cells were present; at the metestrus stage both cornified epithelial cells and leukocytes were present; and at the diestrus stage primarily leukocytes were visible [20]. Control mice were collected at the diestrus stage of the estrous cycle and stage of cycle was unascertainable for PCOS mice as these mice did not cycle.

### Collection and processing of ovaries

The present study was completed using bio-banked ovarian tissue samples from control and PCOS mice from our prior study [20]. Data on body weight/fat, metabolic (cholesterol, triglycerides, insulin tolerance test), hormonal (FSH, LH, testosterone) and reproductive/ovarian parameters have been previously published [20]. Dissected ovaries were weighed and fixed in 4% paraformaldehyde at 4 °C overnight and then stored in 70% alcohol until histological processing [20]. Ovaries were then dehydrated in alcohols, cleared in xylene and embedded in paraffin. A total of *n* = 6 control ovaries and *n* = 6 PCOS ovaries (out of the 8 ovaries available for control mice and 9 ovaries for PCOS/DHT mice) were assessed in the present study. Paraffin-wax embedded ovarian tissue from the DHT-induced PCOS and control mice was sectioned at 4 μm thickness and mounted onto 3-aininopropyltricttiosilane-coated glass slides, then dried overnight in a 50 °C incubator or incubated at 60 °C for 2 h.

### Expression of PAI-1, tPA, uPA, plasminogen and plasmin by immunohistochemistry

We used immunohistochemical detection of PAI-1, tPA, uPA, plasminogen/plasmin and plasminogen in sectioned tissue of both PCOS and control mice. We were unable to identify an antibody to plasmin alone. Immunohistochemistry (IHC) was initially performed on sections of normal mouse tissue known to contain PAI-1, tPA, uPA, plasmin and plasminogen; namely liver for PAI-1, plasminogen/plasmin and plasminogen only, brain for tPA and kidney for uPA (positive control tissues) to determine ideal working conditions. A negative control (to test the effects of non-specific binding of the secondary antibody) was also tested with each marker by omitting the primary antibody. PCOS and control ovarian mouse sections were stained simultaneously for each marker in order to avoid variability due to technical issues. Commercially available primary antibodies that have been shown to work in IHC applications on paraffin-embedded sections (IHC-P) were used for all markers with the exception of the plasminogen only antibody that was shown to work in immunocytochemistry techniques and it was hypothesized that it would also work in IHC. We confirmed this through initial IHC staining of positive and negative controls followed by PCOS and control ovarian tissue.

Slides were deparaffinised in xylene and rehydrated in graded alcohol. Antigen retrieval was performed in a pressure cooker containing sodium citrate buffer (pH 6.0) for 20 min for all markers except for PAI-1 where only 10 min incubation was used and uPA where Tris EDTA buffer (pH 9.0) was used instead of sodium citrate. Endogenous peroxide activity was quenched by 3% H_2_O_2_ in water for 15 min. Non-specific binding was reduced using 1% normal goat serum (ABC Kit VECTASIN). The slides were incubated with the primary antibody at room temperature for either 90 min for PAI-1 (rabbit polyclonal antibody to mouse PAI-1, ABCAM: ab28207) (1:250), uPA (rabbit monoclonal antibody to mouse uPA, ABCAM: ab133563) (1:150), tPA (rabbit polyclonal antibody to mouse tPA, ABCAM: ab28208) (1:200) and plasminogen/plasmin antibodies (rabbit polyclonal antibody to mouse plasminogen/plasmin, Novus Biologicals: NBP2–19859) (1:300) or overnight at 4 °C for the plasminogen only antibody (rabbit polyclonal antibody to mouse plasminogen, Novus Biologicals: NB300–544) (1:100). Slides were then incubated with the secondary antibody (ABC Kit VECTASIN Anti-rabbit IgG biotinylated, affinity purified anti-immunoglobulin) for 30 min for all antibodies except tPA where this step was omitted (as the primary antibody to tPA was biotinylated). 3,3′-diaminobenzidene, DAB (Vector ImmPACT DAB) was used as a chromagen for 2–3 min and haematoxylin was used for counterstaining.

### Follicular classification and enumeration

When describing the expression/distribution of fibrinolytic/proteolytic markers in murine PCOS and control whole ovaries (Table 1), follicles were classified as either small or large. The small follicles described typically represented primordial and primary follicles while the large follicles included secondary and Graafian follicles. The follicle classification system used for analyzing/quantifying individual sized follicles for PAI-1 expression in PCOS and control ovaries (Table 3) was based on the system used by Myers et al [23]. Briefly, primordial follicles consisted of an oocyte surrounded by flattened granulosa cells; primary follicles consisted of an oocyte surrounded by one layer of cuboidal granulosa cells; small preantral follicles contained an oocyte with 1.5–2 layers of cuboidal granulosa cells; large preantral follicles were classified by having an oocyte surrounded by more than two and up to five layers of cuboidal granulosa cells; small antral follicles contained an oocyte surrounded with more than five layers of cuboidal granulosa cells, and/or one or two small areas of follicular fluid whereas large antral follicles contained a single large antral cavity, and preovulatory follicles possessed a single large antrum and an oocyte surrounded by cumulus cells at the end of a stalk of mural granulosa cells [21, 23]. These follicles were further grouped into: small follicles (primordial and primary follicles), medium or preantral follicles (small and large preantral follicles), large or antral follicles (small and large antral follicles) and preovulatory follicles.

### Image analysis

Sections were scanned into digital format using an Olympus Microscope and Slide Scanner (Olympus VS-ASW 2.9, Tokyo, Japan) keeping the (light) exposure time constant for all slides (930 μs). Sections were examined and the distribution of staining in the ovary (PCOS or control) for each marker was determined by an experienced operator (DP) blinded to the identity of the ovarian sample/tissue type (Table 1). Digital image analysis and processing of each scanned slide was performed next using the Olympus cellSens software (Olympus cellSens Dimension Desktop 1.16, Tokyo, Japan), ‘Count & Measure’ functionality that allows selection of a region of interest and cell/region counting capabilities. Intensity analysis was used to determine intensity of (brown) staining in each of the sections. Positive thresholds (of intensity of staining) were set for each marker on the positive control tissues. The positive thresholds for each marker were then applied to the negative control tissues to ensure negative/no detection of staining for the thresholds set. Mouse PCOS and control ovarian tissues were evaluated with the set positive intensity threshold of staining of each marker. Initially the whole ovary for both PCOS and controls was selected as the region of interest (ROI). Overall ovarian staining (Sum Area, μm^2^), percentage of ovarian staining (Area Fraction ROI, %) and intensity of this staining (Mean Colour Intensity) was investigated and compared in PCOS and control ovaries for each plasminogen system marker (Table 2). Overall follicular staining, percentage of follicular staining and intensity of this staining was also investigated and compared for PAI-1 within PCOS and control ovarian follicles in small follicles, preantral follicles, antral follicles and preovulatory follicles (Table 3). Image analysis and processing was performed by an operator (GB) blinded to the identity of the ovarian sample/tissue type (ie PCOS or controls).

### Statistical analysis

Image analysis parameters are presented as mean values ± SD or mean values ±SEM. Statistical analysis was performed using Stata15 (Statacorp, College Station, TX) and SPSS for Windows 22.0 software (SPSS, Inc., Chicago, USA) with statistical significance *p ≤ 0.05. Data was assessed for normality and log transformed where appropriate. Results are presented for a total of 12 mice (*n* = 6 control mice; n = 6 DHT-induced PCOS mice). One ovarian section was selected and assessed from each mouse (i.e. one section per mouse) for every plasminogen system marker evaluated (i.e. 6 ovarian sections from all control mice and 6 ovarian sections from all DHT-induced PCOS mice for PAI-1; alike for the remaining 4 markers). Differences between PCOS and control ovarian IHC staining for all markers were assessed using Student’s *t*-test for normally distributed data and the Wilcoxon-Mann Whitney test for non-normally distributed data. Similarly, Student’s *t*-test was used to compare PAI-1 IHC staining between different sized follicles in PCOS and control ovaries. Further analysis to examine differences in PCOS and controls on the outcomes, t-tests adjusting for potential clustering were performed and Intracluster correlations (ICC) reported.

To further examine the association between the main outcomes of interest using PCOS status as a covariate adjusted by follicular stage, linear mixed models were used. Potential interaction between PCOS status and follicular size on the outcomes were also tested using the above mentioned statistical method.

## Results

### Ovarian morphology

**Fig. 1:**
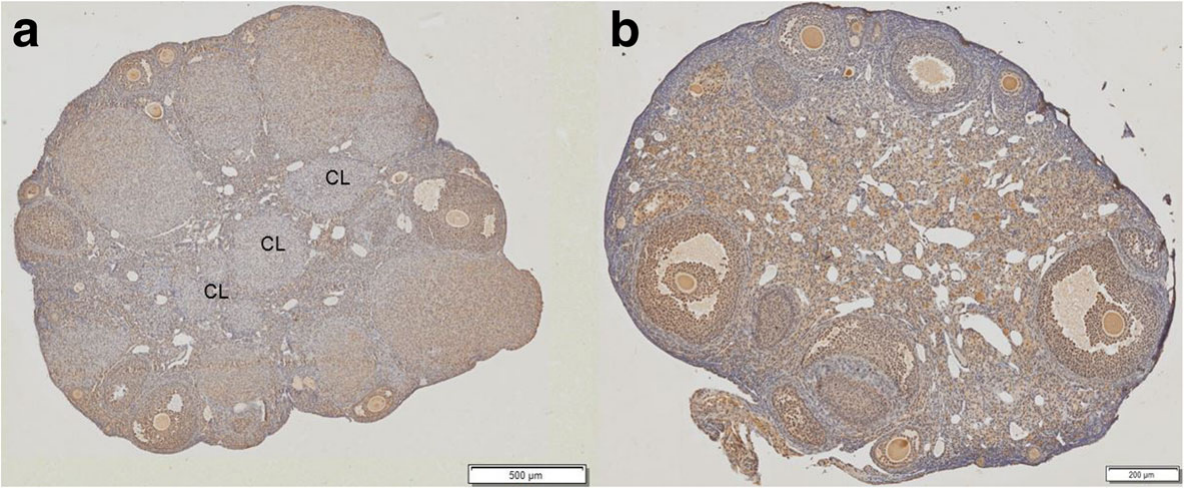
Histological images of normal (control) versus PCOS mouse ovaries. Images demonstrate (**a**) normal mice (controls) ovarian features in the mouse model used including the presence of a corpora luteal (CL) versus no such feature in the (**b**) PCOS mice ovary where mice are acyclical. Several (but not all) representative CL shown in the image. The scale bar represents 500 μm for A and 200 μm for B

Ovaries from DHT-induced PCOS female mice exhibited the classic polycystic ovary morphology with a complete absence of corpora lutea, indicating anovulation. Control ovaries, on the other hand, displayed numerous corpora lutea consistent with recent ovulation (Fig. 1). Moreover, follicles with an atretic cyst-like appearance were observed in ovaries from DHT treated females, but not in control ovaries (Fig. 1).

### Localisation of fibrinolytic/proteolytic markers in murine PCOS and control ovaries

Expression and localization of PAI-1, tPA, uPA, plasminogen/plasmin and plasminogen only in experimental mouse ovaries was investigated.

#### Expression of PAI-1 in mouse PCOS and control ovaries

**Fig. 2:**
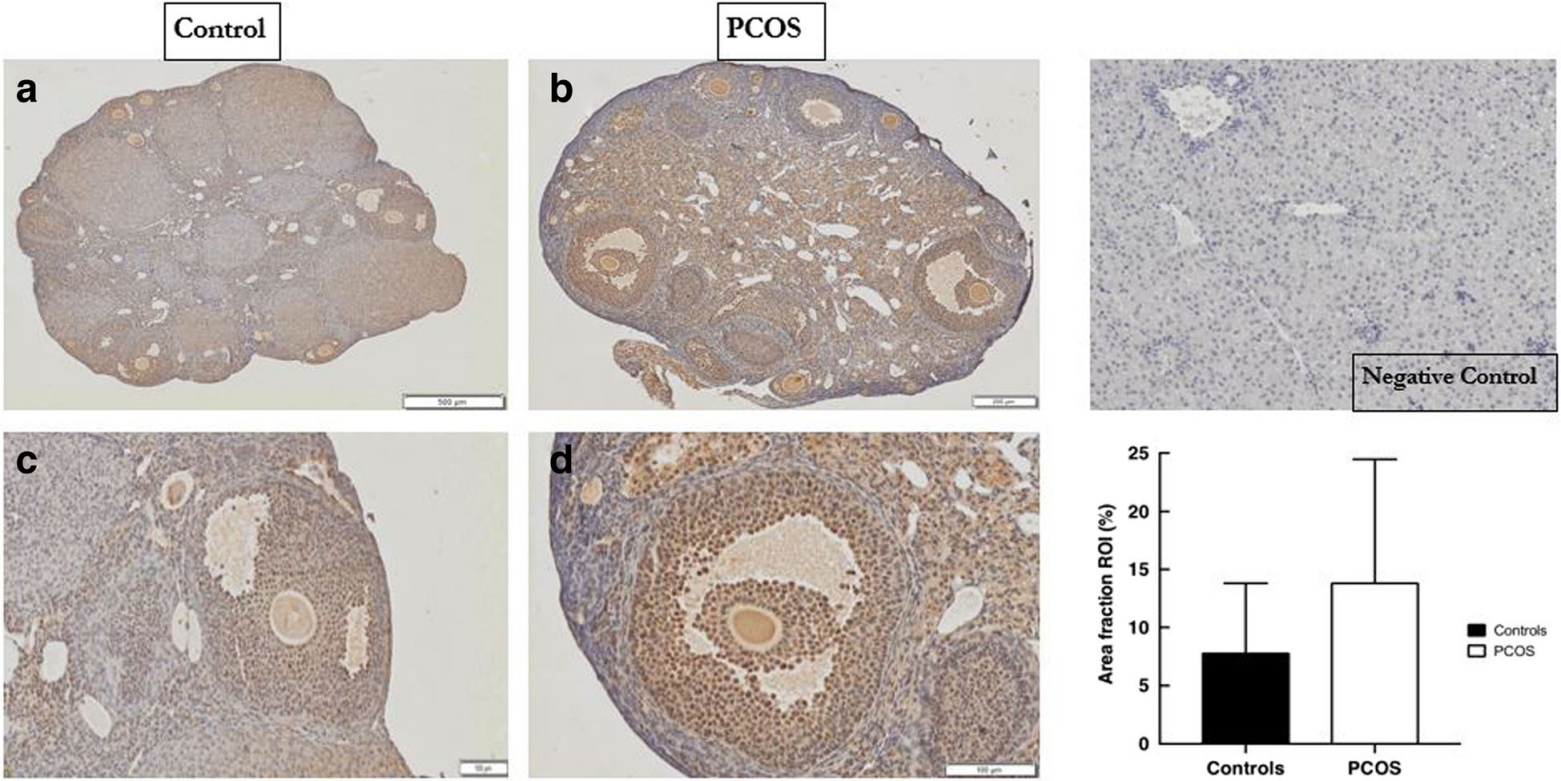
Expression of PAI-1 in normal and PCOS mouse ovaries. Left: Representative histological images are from (**a**, **c**) normal (control) or (**b**, **d**) PCOS ovaries stained with an anti-PAI antibody. The scale bar represents 500 μm for A, 200 μm for B and 100 μm for C & D. Right: Top: Negative control on mouse liver and Bottom: Graphical representation comparing the percentage of ovarian staining (Area Fraction ROI) for PAI-1 between normal and PCOS ovaries for 6 replicates of each experimental group (mean ± SD)

In the non-PCOS mouse (control) ovaries, PAI-1 was predominantly found in the granulosa cells (GC), with greatest expression present in large follicles located mainly in the periphery of the ovary (Table 1 and Fig. 2 a & c). PAI-1 was also observed in the follicular fluid (FF) and in the stroma with only scant amounts detected in the small (central) follicles and theca cells (TC) (Fig. 2 a & c). In the PCOS mouse ovaries, PAI-1 was expressed throughout the ovary. Similar to controls, PAI-1 was also abundant in the GC, and noted in the FF, stroma and large follicles with low amounts observed in the small follicles and TC of PCOS ovaries (Table 1 and Fig. 2 b & d).

**Table 1:** Expression and distribution of the plasminogen system markers in control and PCOS mice ovaries

	Granulosa cells		Theca cells		Large follicles		Small follicles		General distribution	
	Con	PCOS	Con	PCOS	Con	PCOS	Con	PCOS	Con	PCOS
PAI-1	+	+	–	–	+	+	–	–	P	T
tPA	–	–	–	–	–	–	–	–	A	A
uPA	+	+	–	–	+	+	+	+	P	P
Plasminogen/Plasmin	+	+	–	–	+	+	–	–	P	P
Plasminogen only	+	+	+/−	+	+	+	–	+	T	T

‘+’ = expression of marker; ‘-’ = marker absent or in low levels. PAI-1, plasminogen activator inhibitor-1; tPA, tissue plasminogen activator; uPA, urokinase plasminogen activator; Con, controls; P, peripheral; C, central; T, throughout; A, absent. The small follicles described typically represent primordial and primary follicles while the large follicles include secondary and Graafian follicles

#### Expression of plasminogen activators: uPA and tPA in mouse PCOS and control ovaries

**Fig. 3:**
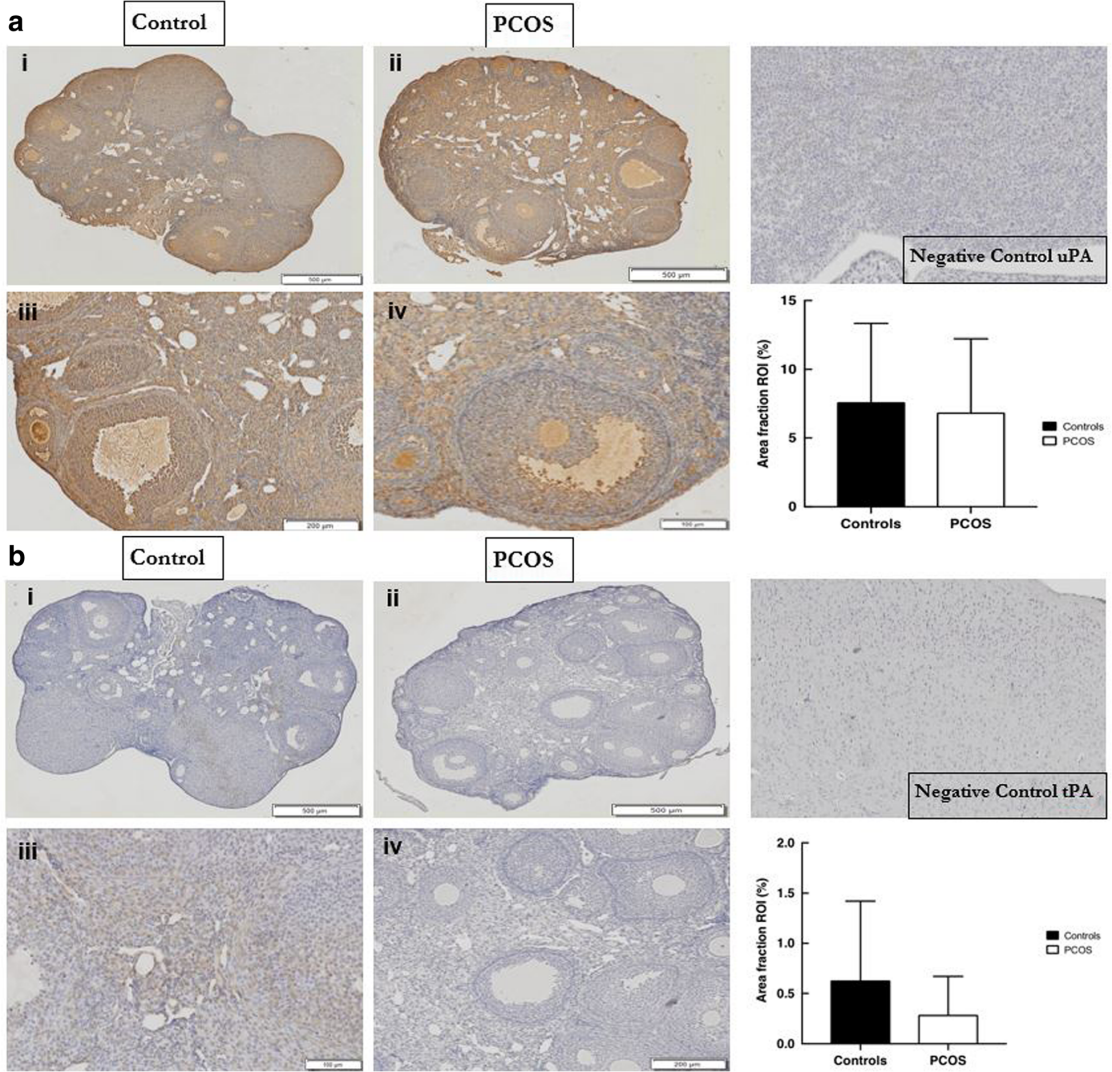
Expression of plasminogen activators uPA and tPA in normal and PCOS mouse ovaries. Left: Top: (**a**) uPA and Bottom: (**b**) tPA. Representative histological images are from normal (control) (Ai, Aiii, Bi, Biii) or PCOS ovaries (Aii, Aiv, Bii, Biv) stained with an (**a**) anti-uPA or (**b**) anti-tPA antibody. The scale bar represents 500 μm for Ai, Aii, Bi & Bii, 200 μm for Aiii & Biv and 100 μm for Aiv & Biii. Right: Top: Negative control for uPA on mouse kidney and graphical representation comparing the percentage of ovarian staining (Area Fraction ROI) for uPA between normal and PCOS ovaries for 6 replicates of each experimental group (mean ± SD) and Bottom: Negative control for tPA on mouse brain and graphical representation comparing the percentage of ovarian staining (Area Fraction ROI) for tPA between normal and PCOS ovaries for 6 replicates of each experimental group (mean ± SD)

Urokinase plasminogen activator (uPA) was present mainly in the periphery of control ovaries as well as in the stroma and GC (Table 1 and Fig. 3 Ai & Aiii). This marker was mainly detected in the large follicles however it was observed in some small follicles but only in scant amounts in the TC and FF (Fig. 3 Aiii). A similar pattern was observed in the PCOS ovaries, where uPA was predominantly observed in the periphery; and was abundant in the GC, as well as in the stroma (mainly in the centre of the ovary), large and small follicles with only small amounts observed in the TC and FF (Table 1 and Fig. 3 Aii & Aiv).

The other PA, tPA, was not observed in either the PCOS or control ovaries (Table 1 and Fig. 3 b), only light staining was observed in the centre of the control ovaries and the stroma, small follicles and GC (Fig. 3 Bi & Biii).

#### Expression of plasminogen/plasmin or plasminogen alone in mouse PCOS and control ovaries

**Fig. 4:**
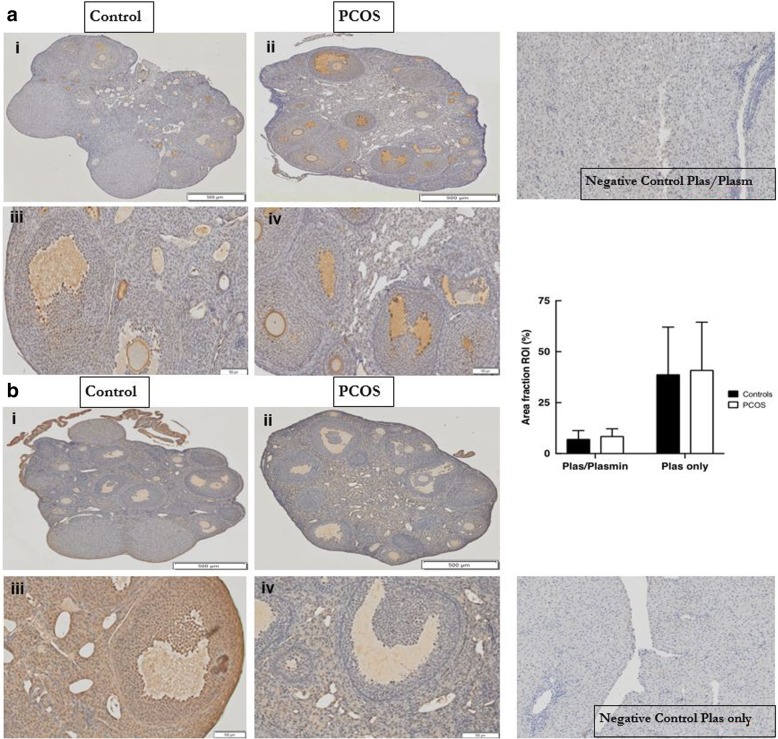
Expression of plasminogen/plasmin (NBP2–19859) or plasminogen alone (NB300–544) in normal and PCOS mouse ovaries. Left: Top: (**a**) plasminogen/plasmin (NBP2–19859) and Bottom: (**b**) plasminogen alone (NB300–544). Representative histological images are from normal (control) (Ai, Aiii, Bi, Biii) or PCOS ovaries (Aii, Aiv, Bii, Biv) stained with an (**a**) anti-plasminogen/plasmin or (**b**) an anti-plasminogen only antibody. The scale bar represents 500 μm for Ai, Aii, Bi & Bii and 100 μm for Aiii, Aiv, Biii & Biv. Right: Top: Negative control for plasminogen/plasmin on mouse liver; Middle: Graphical representation comparing the percentage of ovarian staining (Area Fraction ROI) for plasminogen/plasmin and plasminogen alone between normal and PCOS ovaries for 6 replicates of each experimental group (mean ± SD) and Bottom: Negative control for plasminogen alone on mouse liver

As there was no specific plasmin antibody that could be used for IHC staining we used one which detected both plasminogen/plasmin as well as one that only detected plasminogen in this study. Differences in the staining between these two antibodies would most likely be due to that of plasmin alone. Plasminogen/plasmin (NBP2–19859) was present mainly in peripheral follicles of control ovaries, with less pronounced staining observed in central (small) follicles (Table 1 and Fig. 4 Ai & Aiii). It was present in abundance in GC and FF and also in the stroma. Weak staining was observed in blood vessels as well as in the antrum and TC of control ovaries (Fig. 4 Ai & Aiii). In PCOS ovaries plasminogen/plasmin was mainly observed in the periphery with a similar pattern of distribution in the GC, FF, stroma, large follicles, antrum, blood vessels and TC compared to controls (Table 1 and Fig. 4 Aii & Aiv).

The plasminogen antibody NB300–544 detects only plasminogen but not plasmin. Plasminogen was detected throughout control ovaries, mainly present in the GC and FF (Table 1 and Fig. 4 Bi & Biii). It was also observed in large follicles and sometimes seen in TC while low levels were observed in small follicles, the stroma, antrum and cumulus oophorus of control ovaries (Fig. 4 Bi & Biii). In the PCOS ovaries plasminogen was abundantly present in the FF, and was observed in the TC and GC, as well as in small and large follicles, with low levels in the stroma (Fig. 4 Bii & Biv).

### Comparisons of fibrinolytic/proteolytic markers in murine PCOS versus control ovaries expression using immunohistochemistry

It is well accepted that human PCOS ovaries are generally larger in size and volume compared to non-PCOS ovaries. When performing initial image analysis of the whole ovary, we noted a significant difference in the average ovarian sizes between PCOS and control mice as reflected in the average area ROI (*p* = 0.01). As a result of this we investigated the % staining of the ovaries from control and PCOS mice (Area Fraction ROI) of each marker. Overall, we did not observe any significant difference in the staining for PAI-1, tPA, uPA, plasminogen/plasmin or plasminogen evaluated for either mean total area of IHC staining (Sum Area), percentage of staining (Area Fraction ROI) or average intensity of staining (Mean Colour Intensity Value) of whole ovaries between PCOS and controls (Table 2; *p* > 0.05).

**Table 2:** Comparisons of whole ovarian IHC staining of markers assessed in PCOS and control mice ovaries

Marker	PCOS/ Controls	*N*	Mean Total Area (Sum Area) (μm^2^)(× 10^3^)	Percentage of Staining (Area fraction ROI) (%)	Average Intensity of Staining (Mean Colour Intensity)
PAI-1	Controls	6	281 ± 264	7.86 ± 5.96	178.27 ± 6.79
	PCOS	6	323 ± 266	13.90 ± 10.57	177.07 ± 8.40
tPA	Controls	6	17 ± 22	0.63 ± 0.79	172.90 ± 6.02
	PCOS	6	8 ± 10	0.29 ± 0.38	169.73 ± 4.20
uPA	Controls	6	227 ± 174	7.61 ± 5.72	164.58 ± 6.57
	PCOS	6	155 ± 125	6.86 ± 5.37	163.43 ± 4.04
Plasminogen/Plasmin NBP2–19859	Controls	6	271 ± 183	7.21 ± 4.09	178.95 ± 3.18
	PCOS	6	193 ± 79	8.64 ± 3.53	180.70 ± 6.25
Plasminogen NB300–544	Controls	6	1147 ± 1092	38.93 ± 23.14	165.16 ± 5.09
	PCOS	6	936 ± 646	41.10 ± 23.38	162.72 ± 8.75

Mean ± SD are represented for all values. PAI-1, plasminogen activator inhibitor-1; tPA, tissue plasminogen activator; uPA, urokinase plasminogen activator; ROI, region of interest; N = number of mice ovarian sections or number of mice evaluated (one ovarian section per mouse)

### Comparison of PAI-1 expression in ovarian follicles of PCOS versus control mice using immunohistochemistry

**Fig. 5:**
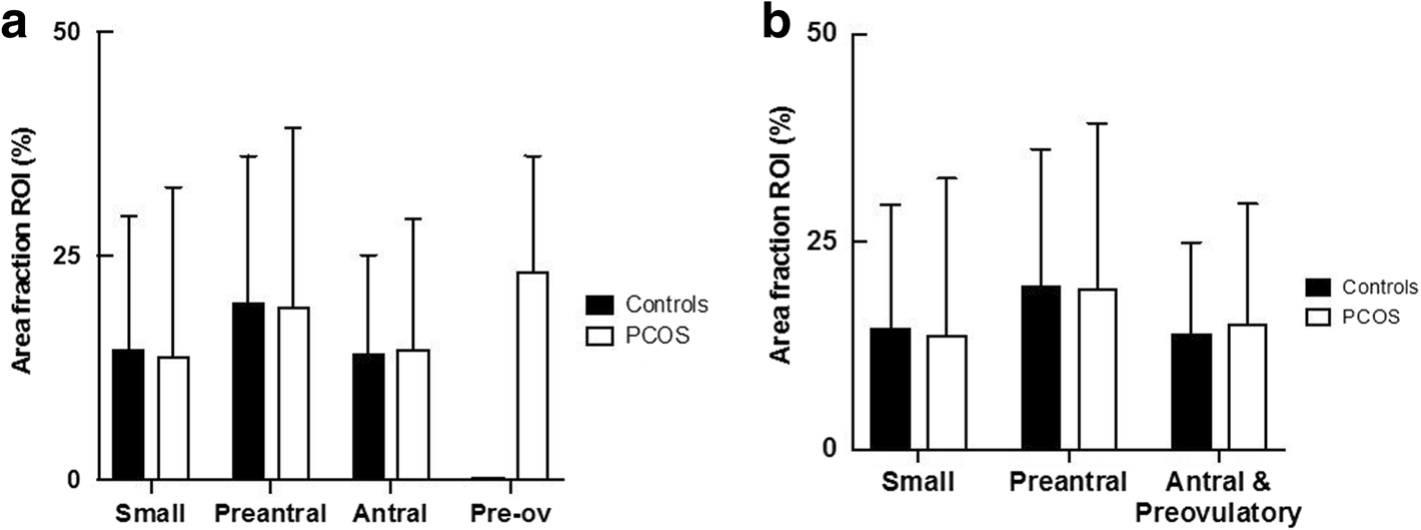
Changes in PAI-1 follicular staining as follicles develop in PCOS and control mouse ovaries. Percentage of PAI-1 follicular staining (Area Fraction ROI) from (**a**) small through to preovulatory follicles (between 41 and 59 replicates for each follicle type/size in each experimental group, with the exception of preovulatory follicles with only 1–3 replicates in each experimental group) and (**b**) with grouped data for antral and preovulatory follicles. Pre-ov = Preovulatory. Error bars represent SD

When we analysed individual sized follicles for PAI-1 expression in PCOS and control ovaries, there was a statistically significant increase in this marker as the follicles developed from small (primordial and primary) through to preantral (medium) follicles in both the control (*p* = 0.03 for % of follicular staining/Area Fraction ROI; *p* = 0.02 for Mean Colour Intensity of follicles) and in PCOS ovaries (p = 0.01 for % of follicular staining; *p* = 0.009 for Mean Colour Intensity of follicles) (Table 3 and Fig. 5 a). A fall in the PAI-1 concentration was then noted from preantral to antral (large) follicles in control ovaries (*p* = 0.009 for % of follicular staining; *p* = 0.004 for Mean Colour Intensity of follicles) though in PCOS ovaries the drop in this marker in these same follicles was not significant/of borderline significance (*p* = 0.09 for Mean Colour Intensity of follicles). A downward trend was noted from antral to preovulatory follicles in control ovaries (% of follicular staining 14.1 ± 11.0 in antral versus 0.24 in pre-ovulatory follicles), however an increase in PAI-1 concentration in these same follicles was observed in PCOS ovaries (% of follicular staining 14.6 ± 14.6 in antral versus 23.3 ± 12.9 in preovulatory follicles) (Table 3 and Fig. 5a), though a statistical analysis could not be completed due to the low numbers of preovulatory follicles. No significant difference was noted in the PAI-1 follicular concentrations in small, preantral and antral follicles in control versus PCOS ovaries (*p* > 0.05). Statistical evaluation could not be made between PCOS and controls in preovulatory follicles due to the low sample size.

**Table 3:** PAI-1 IHC staining in various follicular sizes/stages in PCOS and control mice ovarian follicles

Follicular Size/Stage	PCOS/Controls	Number of Follicles (N)	Mean Total Area (Sum Area) (μm^2^)	*P*-value	ICC*	Percentage of Staining (Area fraction ROI) (%)	*P*-value	ICC*	Average Intensity of Staining (Mean Colour Intensity)	*P*-value	ICC*
Small (Primordial & Primary)	Controls	50	186.54 ± 338.8			14.6 ± 14.9			165.3 ± 43.4		
	PCOS	60	122.7 ± 221.4	0.48	0.17	13.8 ± 18.9	0.92	0.52	155.3 ± 53.4	0.32	< 0.01
Preantral	Controls	44	1505.6 ± 1608.2			19.8 ± 16.4			178.8 ± 7.3		
	PCOS	41	1836.2 ± 2356.4	0.71	0.36	19.4 ± 19.9	0.96	0.61	177.5 ± 11.8	0.82	0.73
Antral	Controls	49	9507 ± 9242			14.1 ± 11.0			175.3 ± 7.23		
	PCOS	53	9092 ± 13,318	0.93	0.41	14.6 ± 14.6	0.94	0.56	171.3 ± 25.8	0.41	0.05
Antral & Preovulatory	Controls	50	9326 ± 9236			13.9 ± 11.0			175.5 ± 7.41		
	PCOS	56	10,132 ± 13,950	0.46	0	15.1 ± 14.5	0.56	0.13	171.2 ± 25.2	0.56	0.92

*ICC = Intra Class Correlation; ROI = region of interest

**Fig. 6:**
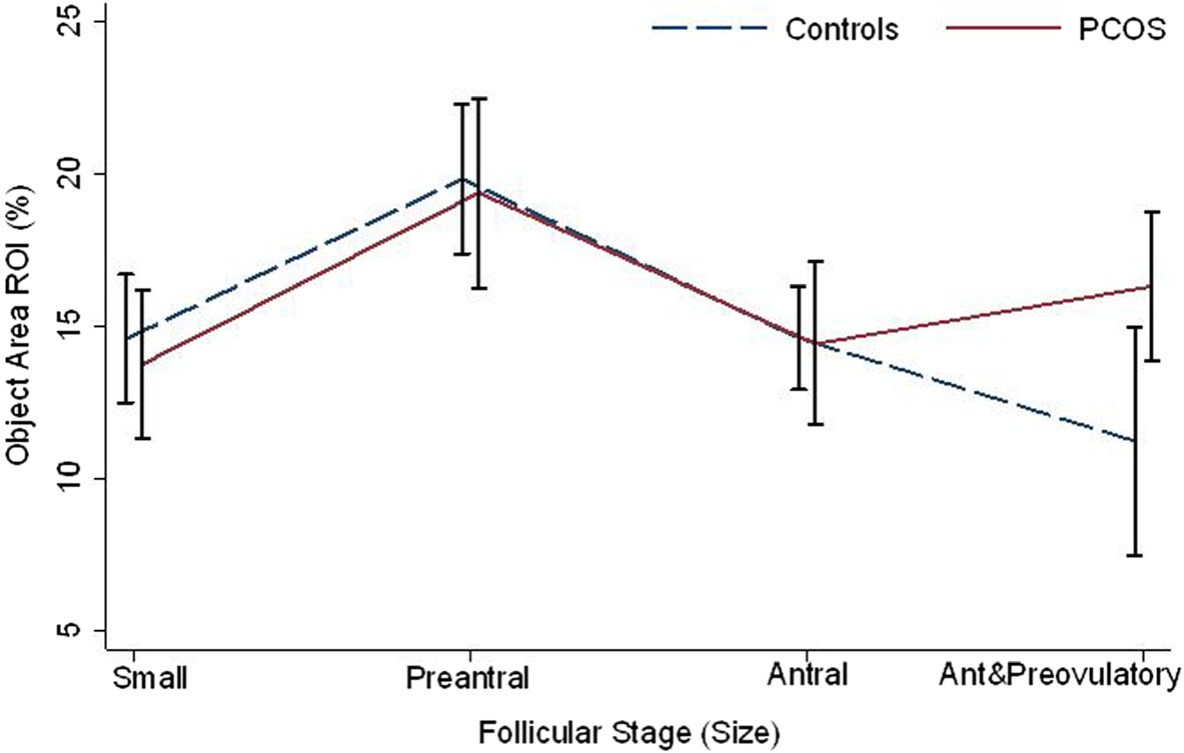
Comparison of PAI-1 follicular expression between PCOS and controls within different follicular sizes/stages. Comparison of PAI-1 follicular expression (Object Area ROI, %) between PCOS and controls within different follicular sizes/stages, from small through to combined antral and preovulatory follicles. Between 41 and 59 replicates for each follicle type/size in each experimental group. Error bars represent SEM

When we grouped the antral and preovulatory follicles together (to increase sample size/follicle numbers), we noted a statistically significant difference when comparing medium sized follicles (preantral) and grouped antral and preovulatory follicles for % of follicular staining for the control ovaries (*p* = 0.02) and no significance difference in PCOS (*p* = 0.11) (Table 3 and Fig. 5 b). Figure 6 displays the changes in PAI-1 expression (%) within different follicular sizes/stages for PCOS and control ovarian follicles, noting similar expression/trends in small through to large follicles for the 2 experimental groups however differential expression/trend for the grouped antral and preovulatory stage.

Further analysis to examine differences in PCOS and controls on the outcomes indicated that there were no statistically significant differences between PCOS and controls for the three outcomes (Mean Total Area, Percentage of Staining and Average Intensity of Staining) categorized by follicular stage. A statistical analysis to test the association between the outcomes of interest and the covariates PCOS status and follicular stage revealed no significant differences between PCOS and controls however, there were significant differences between follicular stage with small as the reference category. The test for potential interactions between PCOS status and follicular stage on the outcomes were statistically non-significant (Tables 3 and 4).

**Table 4:** Linear mixed models noting association between the main outcomes using PCOS status as covariate adjusted by follicular stage

Primary Outcomes	β (95% CI)	*P*-Value	*p*-value (interaction)*
Mean Total Area			
Risk Factors			
PCOS	514 (− 2821 to 3849)	0.76	NS
Follicular type			
small	Ref		
medium	1384 (−500 to 3269)	0.15	
large	10,765 (8774 to 12,757)	> 0.001	
large & preovulatory	6974 (4008 to 9941)	> 0.001	
Percentage of Staining			
Risk Factors			
PCOS	0.28 (−14.1 to 14.0)	0.96	NS
Follicular type			
small	Ref		
medium	5.98 (2.75 to 9.22)	< 0.001	
large	−0.37 (−3.82 to 3.07)	0.83	
large & preovulatory	4.5 (−0.70 to 9.78)	0.09	
Average Intensity of Staining			
Risk Factors			
PCOS	−5.4 (−12.74 to 1.9)	0.15	NS
Follicular type			
small	Ref		
medium	18.02 (8.9 to 27)	> 0.001	
large	12.05 (2.7 to 21.4)	0.25	
large & preovulatory	−4.4 (−30.9 to 22.0)	0.74	

*Test of possible interaction between PCOS status and follicular size on the three outcomes. NS = not significant

## Discussion

To our knowledge, this is the first study that has comprehensively looked at the expression and distribution of fibrinolytic/proteolytic markers and the plasminogen system in a PCOS model. Our novel findings showed a lack of tPA expression in the ovaries of both control and PCOS mice, however the presence and potential role of the fibrinolytic/proteolytic markers PAI-1, uPA, plasminogen and plasminogen/plasmin were demonstrated in the physiological and pathological ovaries of healthy controls and PCOS mice, respectively.

In the control ovary PAI-1 was predominantly observed in the GC, present also in large follicles mainly in the periphery of the ovary, in FF and in the stroma. In the rat, the TC are predominantly responsible for the production of PAI-1 with no synthesis from the oocyte (into the FF); the GC may also express PAI-1 however this will correlate with the stage of the ovulation cycle to ensure appropriate proteolytic (plasmin) activity with each stage [12, 24]. In our study, at the diestrus stage of the cycle, the GC seem to be involved in the production of PAI-1 in mice. In pigs and rats the distribution of PAI-1 immediately prior to ovulation was lower in peripheral/large follicles and highest in small/central follicles to assist in ovulation and follicular wall rupture of mature follicles [11, 25]. As the control mice in our study were in the diestrus stage of the cycle a different pattern was noted, which may be related to differential proteolytic needs at this stage of the cycle (i.e. less needs for a greater plasmin activity) or the differences noted may be related to inter-species differences. In the PCOS ovary PAI-1 was found throughout the ovary, unlike the peripheral distribution observed in the controls. It was abundant in the GC, noted in FF, stroma and large follicles. This pattern of distribution in the PCOS mice may relate to their acyclical nature and the presence of predominantly ‘arrested’ follicles that do not go on to ovulate. As PAI-1 is present in follicles approaching ovulation, and as its levels drop immediately prior to ovulation in the preovulatory follicles, an even ovarian distribution of PAI-1 in the PCOS mice may relate to the presence of follicles in various stages of development along with a reduced number of preovulatory follicles. Our results are similar to that reported by Devin et al who assessed transgenic PAI-1 overexpressing mice that went on to develop multicystic ovaries, in contrast to the wild-type controls [11]. They observed that PAI-1 was abundant throughout the ovaries of the transgenic mice, and was expressed strongly in the GC of developing follicles (and in the thickened tunica, cyst lining and hypertrophied theca) [11]. Studies in humans also observed the presence of PAI-1 in the granulosa cell compartment but also in the TC in both non-PCOS and PCOS ovaries [16]. Devin et al observed PAI-1 to be localised only on the GC lining cystic structures and in atretic follicles of females with PCOS and was absent/observed in scant amounts in human controls [11]. Neither of these research groups however stated the stage of the oestrous cycle of control women and confirmation of elevated androgen levels for all PCOS participants was not undertaken in the study by Atiomo et al [16].

When analysing and quantifying marker expression using image analysis, we initially selected the whole ovary, as the region of interest for all five markers. This is as a result of the lack of published data assessing the plasminogen system in the PCOS ovary. When we compared the mean total amount of overall ovarian IHC staining (Sum Area), the mean total percentage of ovarian staining (Area Fraction ROI) and the average intensity of this staining (Mean Colour Intensity Value) for PAI-1 in PCOS and controls, we found no statistically significant differences between the two groups. This however does not exclude a potential difference in the expression of the marker between PCOS and controls within specific ovarian structures (ie follicles, cells, etc) or at other time-points of ovulation. Our results are however consistent with the study by Atiomo *et at* who also noted overall more PAI-1 was detected in PCOS ovaries but this was not statistically different to controls [16].

We then further investigated and quantified PAI-1 concentrations in developing ovarian follicles in both experimental models. As hypothesized, we found a statistically significant increase in PAI-1 as follicles developed from small through to preantral follicles in both control and PCOS ovaries. This increased expression may be necessary to ensure these follicles remain intact and no premature follicular (wall) rupture takes place. A reduction in PAI-1 expression was observed in both mouse models as follicles developed from preantral through to antral stages, though this was only borderline statistically significant in PCOS ovaries. PAI-1 expression continued to fall in controls from antral through to preovulatory follicles, likely to ensure a high plasmin activity required for follicular rupture immediately preceding ovulation. Conversely, an increase in PAI-1 was noted in PCOS ovaries as follicles developed through these same ovulatory sizes/stages. This may in turn prevent conversion of plasminogen to active plasmin, following from an inhibition of PA by PAI-1, and ultimately a lack of follicular wall breakdown and ovulation in the pathological PCOS ovary. As a low sample size of preovulatory follicles were assessed in PCOS and control ovaries (due to the low numbers of such follicles at the diestrus stage of the cycle in the latter and owing to the acyclical nature of PCOS ovaries in the former) we performed further statistical analyses on grouped data. We also noted a statistically significant difference in PAI-1 concentrations in medium (preantral) versus grouped antral and preovulatory follicles of controls though not significant in these same follicles in PCOS ovaries. Evaluating and comparing the trends between PCOS and control ovaries in PAI-1 expression (Object Area ROI, %) as follicles developed, similar expression/trends were noted in small through to large follicles for the 2 experimental groups however differential expression/trend for the grouped antral and preovulatory size/stage was observed; PCOS mice noted an increased trend in the latter ovarian follicles/stage while a predominantly downward trend was observed for the control mice (Fig. 6).

There were similar patterns of expression and distribution of the plasminogen activator uPA in PCOS and controls with the marker being predominantly present in the periphery of the ovary and in the GC and stroma of both control and PCOS ovaries. Image analyses that quantified this PA did not reveal any significant differences in overall ovarian expression between PCOS and controls. The other PA, tPA was not expressed in either of the assessed mouse models. Here we present novel findings on the presence and distribution of uPA in the PCOS mouse ovary, and demonstrate the expression and potential role of this plasminogen activator in normal physiological folliculogenesis and in early ovarian tissue remodeling processes (at the diestrus stage of the cycle) in normal mouse ovaries; we also demonstrate no distorted presence/distribution in PCOS. Under normal physiological function, uPA (and other PA) are thought to play a role in the formation and maintenance of the corpus luteum and tissue remodeling processes post-ovulation in rhesus monkeys and may also do so in mice [11, 25, 26]. A lack of tPA involvement in both mouse models may relate to interspecies differences (in expression of PA) or may relate to the stage of the estrous cycle when mice were assessed.

A similar pattern of distribution of the plasminogen antibody used in the study (that does not distinguish between plasminogen and active plasmin), was noted in PCOS and control ovaries. Plasminogen/active plasmin was predominantly present in the periphery of control and PCOS ovaries. The plasminogen-specific antibody was expressed throughout the control ovaries with strong staining noted in the FF and also observed in the GC and large follicles of both control and PCOS ovaries. TC and small follicles of PCOS ovaries seem to express plasminogen however this was not observed in controls. Image analyses that quantified the plasminogen/active plasmin or plasminogen only markers did not reveal any significant differences in the overall ovarian expression between PCOS and controls. This is novel data showing plasminogen and/or plasmin expression in mice ovaries. In humans, Ambekar et al noted that plasminogen was degraded in the FF of the ovary in women with PCOS [17]. While we had hypothesized that the differences in staining between the plasminogen/active plasmin and plasminogen only antibodies would reveal the activity of plasmin only in the ovarian tissues, we noted overall stronger ovarian staining with the latter when compared to the former antibody. This may be related to differences in the activity of each antibody type to its antigen/s of interest. We have therefore discussed the activity and distribution of plasminogen and plasmin interchangeably/collectively. The differential expression of the plasminogen specific antibody in the small follicles and in TC of PCOS and control ovaries may also infer differential proteolytic activities between the physiological need and actions of normal ovaries and that of the pathological state in PCOS. This disparity in plasminogen expression between PCOS and control ovaries, combined with differential PAI-1 ovarian localisation and expression within developing follicles in the 2 experimental models, as was noted earlier, may be induced by the androgen (DHT) excess in the PCOS model versus that of controls, and suggests androgens may be involved, at least in part, in regulating expression of ovarian fibrinolytic/proteolytic markers and the ovarian plasminogen system.

Limitations of the current study include use of a PCOS mouse model that does not display all features of the human condition, though no animal model has been developed to date which does. The long-term postnally treated DHT PCOS mouse provides the best mouse model for experimental studies of PCOS pathogenesis through replicating extensive ovarian (irregular cycles/acyclicity, oligo−/anovulation, multicystic ovaries, antral follicle arrest, increased follicle atresia, reduced granulosa and increased TC layer thickness), endocrine (reduced progesterone and increased DHT) and metabolic (obesity, adipocyte hypertrophy, dyslipidaemia and presence of steanosis) features of humans affected by the syndrome [20]. Strengths of our study include a comprehensive and novel analysis of the plasminogen system in control and PCOS mouse ovaries, with few or no previous studies undertaken in either animal PCOS models or in human PCOS studies.

## Conclusions

Overall our results demonstrate both the presence of four of the five fibrinolytic/proteolytic markers assessed and note some differential expression of the plasminogen system in the functional mouse ovary and in PCOS. PAI-1 was expressed throughout the PCOS ovary, unlike the peripheral distribution observed in control ovaries and plasminogen was present in the small follicles only in PCOS ovaries but not in the small follicles of control ovaries. This may infer an active role of the proteolytic/fibrinolytic system markers in early ovarian function including in follicular development processes and ovulation as well as differential proteolytic outcomes when compared to the pathological PCOS state. Our results however do not show a statistically significant difference between markers assessed in PCOS versus controls for overall (whole) ovarian expression or in individual sized follicles for PAI-1. These outcomes may be related to the small sample size assessed and the stage of the ovulation cycle of mice. The differential expression of PAI-1 and plasminogen only in PCOS and control ovaries as well as observations of differential PAI-1 patterns as follicles develop may indicate potential differences in proteolytic actions/levels of these markers and further support our earlier conclusions of a possible disparity in underlying causes or effects between ovarian physiological and pathological (PCOS) states. These outcomes may be related to differential androgen (DHT) levels between the two experimental models and infer that androgens may play a role in regulation of expression of ovarian plasminogen system markers.

Further studies with larger samples sizes and evaluating these fibrinolytic/proteolytic markers at different time-points of the oestrous cycle, particularly immediately preceding ovulation, may help to further clarify physiological and any potential pathological roles of these markers in the aberrant ovulation process and in folliculogenesis in PCOS. Such studies may significantly improve our understanding of this very complex condition as well as assist with treatment options for the frequent infertility noted for women with this common syndrome.

## Abbreviations

AR: Androgen receptor
CL: Corpora luteal
CVD: Cardiovascular disease
DHT: Dihydrotestosterone
FF: Follicular fluid
GC: Granulosa cells
ICC: Intra Class Correlation
IHC: Immunohistochemistry
IHC-P: IHC applications on paraffin-embedded sections
PA: Plasminogen activator/s
PAI-1: Plasminogen activator inhibitor 1
PCOS: Polycystic Ovary Syndrome
ROI: Region of interest
TC: Theca cells
tPA: Tissue plasminogen activator;
uPA: Urokinase plasminogen activator
VTE: Venous thromboembolism
